# Characterization of Asphalt Binder and Mixture for Enhanced Railway Applications

**DOI:** 10.3390/ma18143265

**Published:** 2025-07-10

**Authors:** Ilho Na, Hyemin Park, Jihyeon Yun, Ju Dong Park, Hyunhwan Kim

**Affiliations:** 1 Barun Construction Technology, Youngin 16953, Republic of Korea; ihna2209@gmail.com (I.N.); phmin84@naver.com (H.P.); 2 Department of Engineering Technology, Texas State University, San Marcos, TX 78666, USA; yiy1@txstate.edu; 3 Department of Maritime Police and Production System, Gyeongsan National University, Tongyeong 53064, Republic of Korea

**Keywords:** railway, rutting, indirect tensile strength (ITS), tensile strength ratio (TSR), styrenic thermoplastic elastomer (STE), crumb rubber modifier (CRM)

## Abstract

Although asphalt mixtures can be applied to railway tracks due to their viscoelastic properties, caution is required, as their ductility and brittleness are highly sensitive to temperature variations. In recent years, interest in the application of asphalt in railway infrastructure has increased, driven by the development of modified mixtures and the broader availability of performance-enhancing additives. Additionally, evaluation methods for railway tracks should be adapted to account for the distinct loading mechanisms involved, which differ from those of conventional roadways. In this study, the comprehensive properties of asphalt binders, mixtures, and testing methods—including physical and engineering characteristics—were assessed to improve the performance of asphalt concrete layers for potential applications in railroad infrastructure. The results of this study indicate that (1) the higher the performance grade (PG), the higher the indirect tensile strength (ITS) value achieved by the 13 mm mixture using PG76-22, which is higher than that of the PG64-22 mixture. This indicates that higher PG grades and modification contribute to improved tensile strength, beneficial for upper layers subjected to dynamic railroad loads. (2) The tensile strength ratio (TSR) increased from the unmodified mixture to over 92% in mixtures containing crumb rubber modifier (CRM) and styrenic thermoplastic elastomer (STE), demonstrating enhanced durability under freeze–thaw conditions. (3) Wheel tracking test results showed that modified mixtures exhibited more than twice the rutting resistance compared to PG64-22. The 13 mm aggregate mixtures also generally performed better than the 19 mm mixtures, indicating reduced permanent deformation under repeated loading. (4) It was concluded that asphalt is a suitable material for railroads, as its overall characteristics comply with standard specifications.

## 1. Introduction

In general, asphalt mixtures are known to have positive effects such as energy absorption, noise reduction, and economy as materials used for highway pavement [1,2,3]. However, the asphalt mixture is considered sensitive to high temperatures for rail tracks. It is ductile in summer and brittle in winter, so it is relatively easy to damage. However, various additives can improve the properties of asphalt to compensate for these drawbacks. Therefore, interest in the application of asphalt to railway tracks is increasing due to the recent popularization of several additives and the development of modified asphalt mixtures [4,5,6,7].

Research on using asphalt concrete is largely divided into asphalt roadbeds for gravel tracks, concrete tracks, and asphalt tracks (direct track). Many developed countries are introducing a new conceptual asphalt concrete track by applying asphalt materials and directly combining them with sleepers, gravel tracks, and concrete tracks to solve various problems using the asphalt mixture [8,9].

Early track designs consisted of two parallel rails anchored to wooden sleepers, and the sleepers were installed on plain ground. Afterward, to improve the railway track, the gravel track was developed. It suppressed the horizontal and vertical movement of the railway under load and showed an effective reduction in displacement [10]. However, the gravel track also requires frequent maintenance due to the flow and track destruction of the gravel, which is caused by the train load and a decrease in permeability [11,12,13]. Concrete tracks can reduce maintenance costs and secure buckling stability. However, the initial construction cost is high, and repairs are complex when replacing sleepers or disposing of concrete ballast. Asphalt concrete tracks have various cross-sectional shapes to improve riding comfort by reducing noise and vibration and have the advantage of shorter maintenance and traffic opening time compared to concrete [14,15,16]. The disadvantage is that permanent deformation may occur due to temperature sensitivity; hence, it is necessary to manage the air voids through proper mix design considering the railroad load, which is also related to the life cycle. According to a research report by Rail One of Germany, asphalt concrete life expectancy is 60 years, which can be sufficiently improved through the development of special asphalt mixtures.

**Figure 1:**
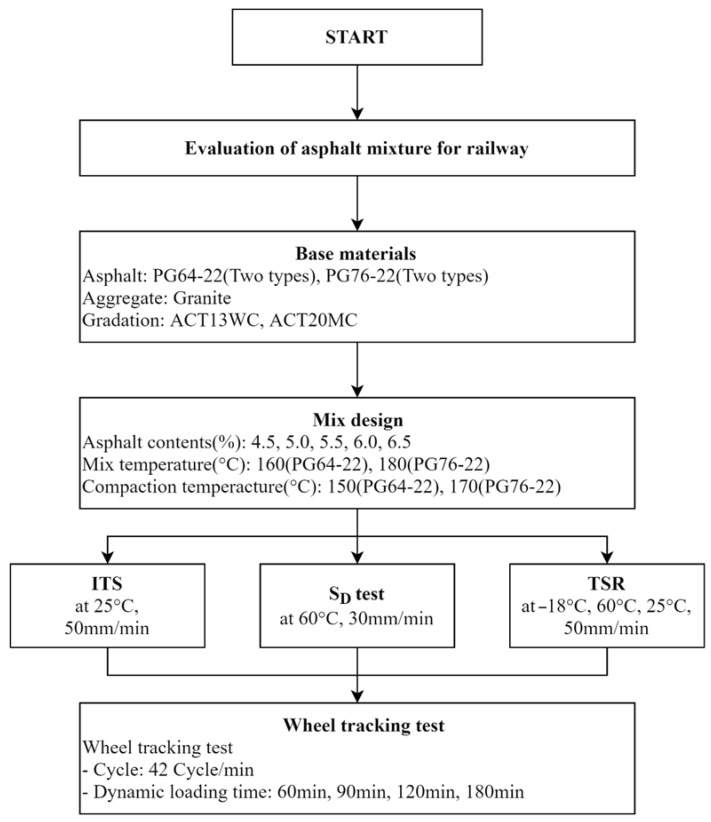
Flow chart of experimental design procedures.

Unlike rigid tracks such as concrete tracks, asphalt concrete (flexible tracks) must be evaluated for resistance to permanent deformation considering the material properties depending on temperature [17,18]. Therefore, the objective of this study is to evaluate the overall characteristics of asphalt binders and mixtures to improve the quality of asphalt concrete layers for railway applications, as shown in Figure 1.

## 2. Materials and Methods

### 2.1. Materials

#### 2.1.1. Aggregate

The aggregate used in this study was granite crushed stone, and the maximum size of the coarse aggregate was 13 mm and 19 mm. Coarse and fine aggregate was utilized after the screening, and limestone powder was used as a filling material (Table 1).

**Figure 2:**
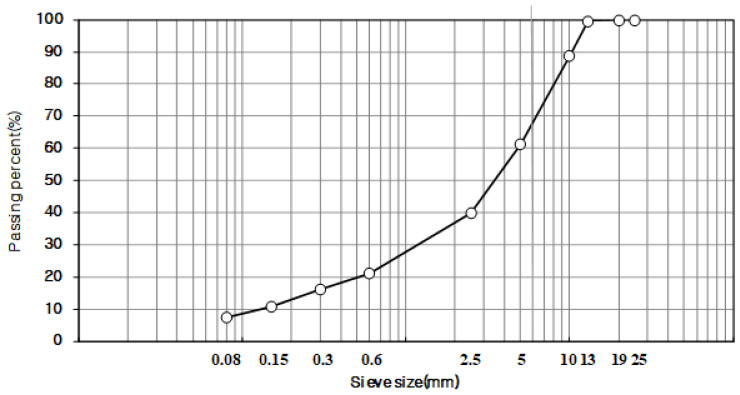
Aggregate gradation chart (ACT13WC).

**Figure 3:**
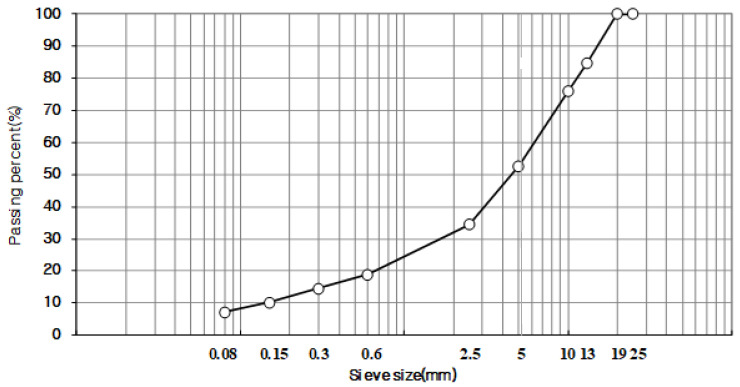
Aggregate gradation chart (ACT20MC).

The mix design used the particle sizes of ACT13WC and ACT20MC, which were researched and developed by the Korea Railroad Research Institute. After years of research on roadbeds using asphalt mixtures, the maximum size of coarse aggregates suitable for asphalt mixtures for railways was selected. Also, the standard for particle size was set by applying the specification standard used during site laying. Through the sieve test, the gradation was determined using the passing mass percentage of each body, satisfying the upper and lower limit standards presented in the specification. Figure 2 and Figure 3 show the grain size distribution curves for 13 mm and 19 mm that were used in this study, respectively.

#### 2.1.2. Polymer-Modified Asphalt (PMA) Binders

This study used two performance-grade (PG) asphalt binders, PG 64-22 and PG 76-22. PG 64-22 has one neat asphalt binder and a 1.5% styrenic thermoplastic elastomer (STE) binder. Also, two different PG76-22 were produced with 5% STE and 8% crumb rubber modifier (CRM). Table 2 briefly explains the labeling and content of the binders.

### 2.2. Methods

#### 2.2.1. Binder Tests

Modified asphalt is required for railroad applications, as it enhances stiffness and resilience to effectively absorb vibrations generated by train operations. To satisfy these requirements, newly developed PG64-22 and PG76-22 asphalt binders were tested to secure structural stability against loads transmitted from panels and wide sleepers. Essential characteristics were evaluated, such as softening point, penetration, ductility, and rotational viscosity. In addition, the dynamic shear rheometer (DSR) and bending beam rheometer (BBR) tests were conducted considering rheology properties.

Rotational viscosity tests were performed at 135 °C and a spindle speed of 20 rpm to assess the workability of the asphalt binder, based on the relationship between fluid shear, spindle radius, and rotation speed. Data was collected for 15 min at 1 min intervals after 30 min of stabilization.

The dynamic shear rheometer (DSR) is test equipment for analyzing the viscoelastic behavior of asphalt binders. Since the behavior of asphalt is affected by time and temperature, it is ideal to measure the effects of time and temperature simultaneously. The DSR test analyzes an asphalt binder’s viscous and elastic behavior characteristics by measuring the complex shear modulus (G*) and phase angle (*δ*). The G*/sin *δ* value is a characteristic value composed of two characteristics, G* and *δ*. A binder with high elasticity has a high value of G*/sin *δ*, and a binder with high viscosity has a low value. G*/sin *δ* represents the stiffness of the binder at a common pavement temperature, so it is called the rutting factor. It is used to evaluate the PG of the asphalt binder. The G*/sin *δ* standard value of the high-temperature grade is 1.0 kPa for non-aging (original) binders, and the binder after short-term aging treatment with a rolling thin-film oven (RTFO) should be 2.2 kPa or more. Additionally, fatigue cracking is addressed by specifying that the binder’s G*·sin δ value must be less than 5000 kPa after aging in the pressure aging vessel (PAV).

The BBR test is used to determine the cracking resistance of asphalt pavements at low temperatures. In the bending beam rheometer (BBR) test, a constant creep load is applied to an asphalt beam using a bending mold at a specified low temperature, which is maintained in a methanol-filled bath. The asphalt beam has a length of 125 mm, a width of 12.7 mm, and a thickness of 6.35 mm, and a load of 100 g is applied to the center of the beam for 240 s to measure the creep stiffness.

#### 2.2.2. Mix Design

**Figure 4:**
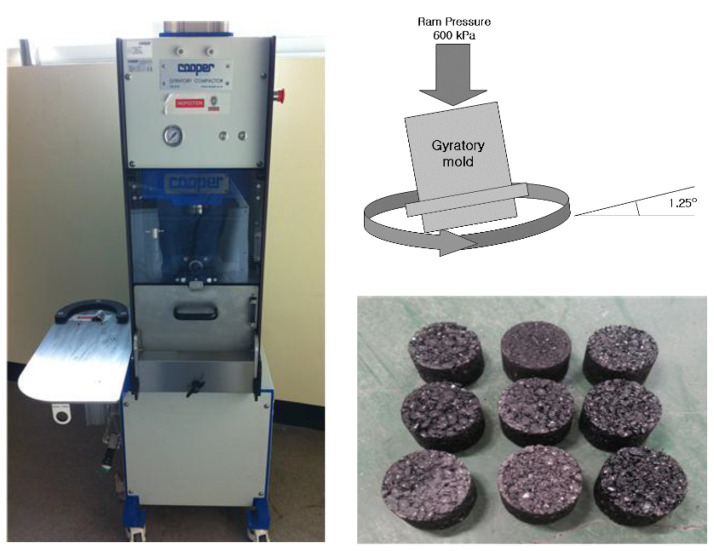
SGC equipment and compactor mechanism: specimen sample.

A Superpave gyratory compactor (SGC) was used, as shown in Figure 4, to determine the optimum asphalt content (OAC) of the asphalt mixture. The control factors for the compaction results are vertical pressure (600 kPa), tilted angle (1.25°), turning compaction speed (30 times/min), and deformation strength (S_D_), and they were used to determine the OAC. The OAC was determined within the range satisfying each standard as voids of 1 to 3%, VFA of 80 to 90%, VMA of 12% or more, and S_D_ of 3.2 MPa or 4.25 MPa or more.

Mixture design was performed for a total of 6 mixtures. One type of aggregate (granite), two maximum coarse aggregate sizes (13 mm and 19 mm), and four types of binders (AP-5, STE, STE5, CRM) were combined. As shown in Table 3, the 13 mm size was applied for all types of binders because there are cases where PG64-22- and PG76-22-grade asphalt mixtures are used depending on site conditions (track type, temperature, train passing tonnage, etc.). However, the 19 mm mix design was performed with only the AP-5 and STE of PG64-22, except for PG76-22 since 19 mm is applied only to the middle layer.

Mixture design was performed using a gyratory compactor, applying the characteristic standard values for asphalt concrete roadways presented in Table 4, based on research by the Korea Railroad Research Institute. In Table 4, the middle layer is named ACT20MC, the surface layer is named ACT13WC, and each asphalt mixture satisfies the presented criteria. In addition, the tensile strength ratio, dynamic stability, and depth of settlement must be evaluated for the surface layer, and the middle layer is the same as the surface except for tensile strength.

#### 2.2.3. Deformation Strength (S_D_)

Deformation strength is a characteristic value that shows a high correlation with the permanent deformation characteristics of asphalt mixtures and is one of the important variables in mix design in South Korea [19]. The deformation strength was calculated using Equation (1) by reading the maximum load (*P_max_*) and the vertical strain (*v*) pressed from the surface at this time in the load–strain curve obtained by applying a load to the specimen at 60 °C [20,21,22].

(1) $$S_{D}=\frac{0.32P_{max}}{(10+{\sqrt{20v-v^{2}})}^{2}}$$

*S_D_*: deformation strength (MPa),

*P_max_*: maximum load (N),

*v*: vertical deformation at maximum load (mm).

**Figure 5:**
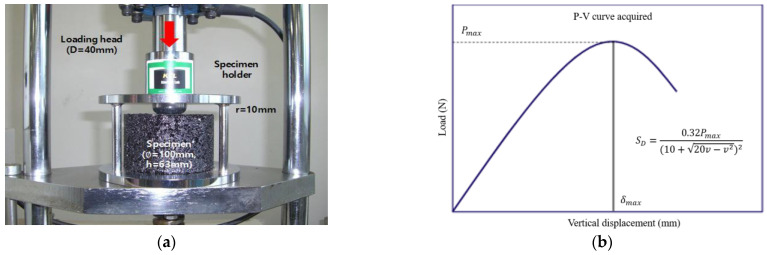
(**a**) Kim test setting in a loading frame; (**b**) load–vertical displacement (P-V) curve after deformation strength test.

The specimen was taken out after being immersed in a water bath at 60 °C for 30 min and quickly put into the Kim test assembly, and a load was applied at a loading speed of 30 mm/min. The maximum load (*P_max_*) and vertical displacement (*v*) were obtained from the curve, and the average value of three S_D_ tests per mixture was used for the analysis. Figure 5a,b show the specimen set in the Kim test assembly and the load–displacement (P-v) curve after the strain test, respectively.

#### 2.2.4. Indirect Tensile Strength (ITS)

Cracks often develop between sleepers and asphalt concrete roadbeds, leading to significant sleeper displacement and track misalignment. These cracks can propagate rapidly due to factors such as impact loads from train operation, traffic volume, climate variations, and train speed. This deterioration may compromise driving stability, the structural integrity of the asphalt concrete, and passenger comfort, as repeated train loads on a single sleeper can influence the behavior of adjacent sleepers. Therefore, to effectively evaluate the performance of railway asphalt mixtures, both tensile strength and tensile strain should be determined using an indirect tensile strength test.

**Figure 6:**
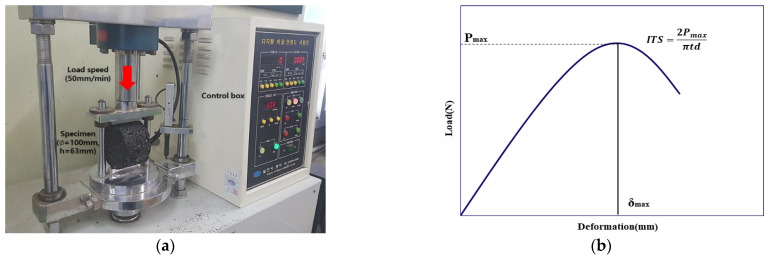
(**a**) ITS test setting in a loading frame; (**b**) load–deformation curve after indirect tensile strength test.

Tensile strength can be applied to predict the crack resistance of asphalt mixtures, so mixtures that withstand high tensile strength at flexural tensile failure can be considered to have excellent crack resistance. Figure 6a shows the indirect tensile strength test conducted after placing the specimen in the test device. Figure 6b shows the load–displacement graph when the load was applied. The ITS at maximum load is given by Equation (2).

(2) $$ITS=\frac{2P_{max}}{\pi td}$$

*ITS* = indirect tensile strength (Mpa),

*P* = maximum load (N),

d = specimen diameter (mm),

t = specimen thickness (mm).

#### 2.2.5. Tensile Strength Ratio (TSR)

Asphalt mixtures lose strength as they are exposed to water. This is mainly due to the peeling of the asphalt film covering the aggregate. In addition, when a mixture containing water freezes, the moisture in the pores expands, causing internal stress. Microcracks are generated by internal stress, thereby reducing the strength of the mixture. This phenomenon appears to be more severe when the bonding force binding to the aggregates in the mixture is weak.

The voids for general roads are targeted at 4%, and the air void for moisture-sensitive roads is obtained from compaction [(1 − 0.96 × 0.96) × 100 = 7.8%] of 96%. To match the value, since the voids for asphalt concrete railroad tracks are less than 3%, the voids suitable for the moisture sensitivity test are compacted to at least 97% of the designed voids [(1 − 0.97 × 0.97) × 100 = 5.9%], so the study was conducted by setting 5 ± 0.5%.

In this study, six specimens were prepared using the freeze–thaw treatment method with air voids of 5 ± 0.5% according to AASHTO T 283, three of which were dry, and the other three were soaked in water (after partial saturation and freeze–thaw). The water immersion-treated specimen was partially saturated, wrapped in plastic, cooled to −18 °C for 1 h, maintained at that temperature for 16 h, then immersed in a 60° C water bath for 24 h, followed by immersion in a 25 °C water bath for 2 h, after which the ITS was measured.

In addition, the three dried specimens were stored at room temperature for the same time as the water immersion-treated specimens, and then placed in a water bath at 25 ± 1 °C for 2 h before ITS measurement. Then, the ITS was measured in the same way. After that, the TSR was calculated using the two tensile strength values obtained from Formula (3).

(3) $$TSR=\frac{{ITS}_{WET}}{{ITS}_{DRY}}$$

*ITS_WET_* = ITS of the water immersion-treated specimen (MPa)

*ITS_DRY_* = ITS of the untreated specimen (MPa)

#### 2.2.6. Wheel Tracking Test

The main cause of permanent deformation was determined to be the shear deformation of the asphalt mixture. Permanent deformation in asphalt concrete pavement can occur in all layers of the pavement, such as the roadbed, subbase layer, base layer, and pavement layer, but permanent deformation is mainly generated on the asphalt surface. This permanent deformation is influenced by the asphalt binder and the aggregate, which account for 88–96% of the asphalt concrete mixture.

In the study, slab specimens of 305 × 305 × 50 mm were manufactured with air voids of 1 to 3% using Cooper’s roller press compactor to perform a wheel tracking test. The prepared specimens were cured at room temperature for 24 h and then conditioned at 60 °C for 6 h prior to testing. The test was conducted at 60 °C according to KS (Korean industrial standards), and the final rut depth was measured by repeating 2520 passes for 60 min with a wheel load of 686 N (70 kg) and a passing speed of 42 times/min. The material of the wheel is steel, the diameter is 200 mm, the width is 50 mm, and the round-trip distance is 200 mm.

Dynamic stability (DS) is expressed as the number of passages that generate 1 mm of vertical deformation in a specimen, and the deformation rate is expressed as deformation per minute. The dynamic stability and strain rate were obtained as shown in Equations (4) and (5).

(4) $$DS_{KS}=42\times \frac{t_{2}-t_{1}}{d_{2}-d_{1}}\times c$$

*DS* = dynamic stability

*d*_1_ = deformation at t_1_ (45 min) (mm)

*d*_2_ = deformation at t_2_ (60 min) (mm)

*c* = 1.0 as correction factor

(5) $$RD=\frac{d_{60}-d_{45}}{15}$$

*RD* = deformation rate

*d*_60_ = deformation at 60 min (mm)

*d*_45_ = deformation at 45 min (mm)

**Figure 7:**
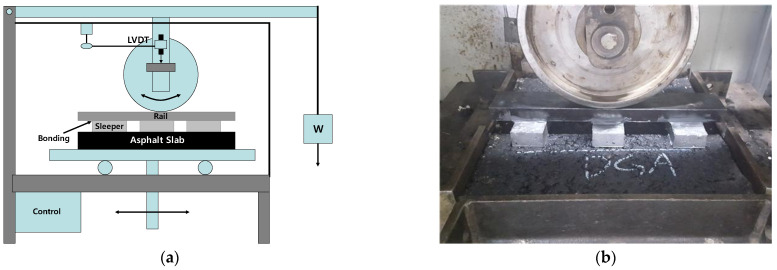
Wheel tracking test equipment for roadway asphalt pavement. (**a**) Illustration of WT test equipment concept; (**b**) illustration of actual MWTT setup.

**Figure 8:**
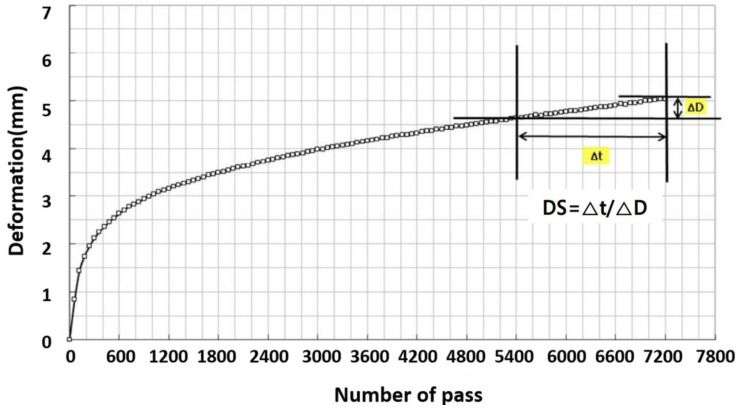
Dynamic stability curve obtained from the wheel tracking test.

Figure 7a illustrates the modified wheel tracking test apparatus used to simulate railroad structures in this study. The device features a stroke length of 200 mm and utilizes steel wheels to closely replicate the material properties of actual train wheels. To simulate realistic railway conditions, a set-up consisting of one rail and three sleepers in contact with the wheel was assembled before testing. Figure 7b presents the experimental set-up prior to initiating the repeated loading test using the modified wheel tracking system. The DS_KS_ values were primarily derived from rut depth measurements taken at 45 min (d1) and at the end of the test (d2). For extended evaluation over a total duration of 180 min (10,800 s), rut depth was measured continuously and analyzed at intervals of 60, 90, 120, and 180 min to assess the long-term performance relevant to railway applications. Figure 8 shows a general graph to calculate the DS_KS_ in this study. Throughout the testing, rut depth was recorded in real time using a linear variable differential transformer (LVDT), synchronized with elapsed time data to ensure accurate monitoring of deformation behavior under repeated loading.

## 3. Results and Discussion

### 3.1. Asphalt Binders

Kinematic viscosity, softening point, penetration, and ductility were measured as rheological characteristics according to the type of asphalt binder, as shown in Table 5. In the results of rotational viscosity at 135 °C, STE5 showed the highest viscosity of 1.8P∙s, and CRM showed the lowest value of 1.0P∙s. In addition, AP-5 and STE showed a difference of 0.01P∙s in rotational viscosity, confirming no significant difference between them.

AP-5 and STE showed the same characteristics in penetration and softening point, and CRM and STE5 showed similar values. However, there is a clear difference according to the amount of modifier, indicating that the modified asphalt of CRM and STE5 has a lower penetration value and a higher softening point than AP-5 and STE. This result is attributed to the increased elasticity rather than the viscosity of asphalt, because CRM and STE5 have relatively high polymer content. Based on these results, it is implied that adding polymers, including PG76-22 base binder, is effective in making more rigid materials with high viscosity and reduced softness.

In this study, both the fundamental physical properties of binders and the viscoelastic properties of the asphalt binder were confirmed using the DSR test to measure the values of G*/sin *δ* and G*∙sin *δ*. In addition, creep stiffness and the m-value were measured using the BBR test to evaluate thermal cracking resistance. Table 5 shows the results of the DSR test for the four types of asphalt binders used at high temperatures, categorized into original and RTFO binders. For the AP-5 and STE binders, the performance at high temperatures was found to be 64. In addition, CRM and STE5 containing polymer modifiers showed a performance of 76. The BBR test was conducted to confirm that the low-temperature grade and all binder grades were measured as −22 through creep stiffness and m-value measurements.

### 3.2. Asphalt Mixture

#### 3.2.1. Mix Design

Four types of asphalt binders (AP-5, STE, CRM, and STE5) were used to determine the optimum asphalt content (OAC) based on mix designs incorporating 13 mm and 19 mm aggregates.

For a coarse aggregate maximum size of 13 mm, the optimum asphalt content (OAC) was 5.2% for AP-5 and 5.1% for STE. The OAC for both CRM and STE5 was determined to be 5.6%. In addition, AP-5 and STE had void ratios ranging from 1 to 3% at 5.0% and 4.9% of OAC for a 19 mm aggregate size. For VMA and VFA, the OAC was finally determined to correspond to about a 2% void ratio, as shown in Table 6 for all six mixtures.

#### 3.2.2. S_D_ and ITS

**Figure 9:**
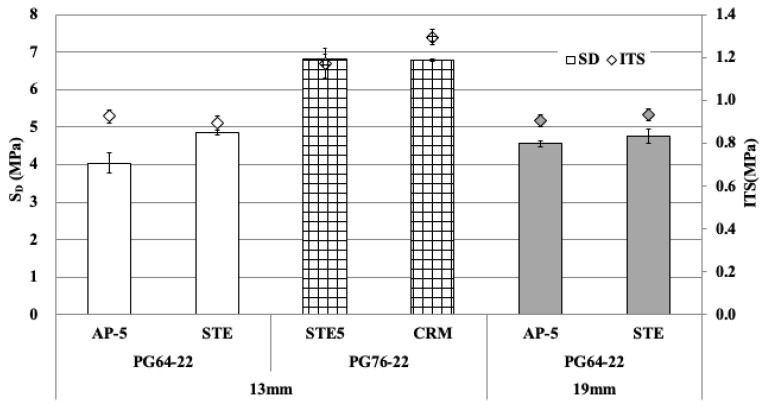
Results for mechanical properties of asphalt mixtures for the asphalt concrete track.

The test specimens were manufactured with the OAC determined through the mix design. Then, the deformation strength and indirect tensile strength were measured using these specimens. The results are shown in Figure 9. As for the deformation strength, an average of 4.44 MPa was measured at a 13 mm mix design in PG64-22. An average of 4.67 MPa was observed for the 19 mm mix design, indicating that the 19 mm performed better, with a difference of about 0.22 MPa. It was found that the optimum asphalt content was relatively low, which resulted in reduced deformation. Consequently, the deformation strength value was slightly higher. All measured values were found to exceed the test criteria.

In the ITS results, the PG76-22 asphalt mixture showed a higher value of about 0.33 MPa than the PG64-22 with the 13 mm mix design. On the other hand, the ITS values for the 13 mm and 19 mm mixes of PG64-22 were about 0.91 MPa on average, showing similarity.

On the other hand, both PMA binders (STE5 and CRM) with 13 mm aggregate showed about 6.8 MPa in the deformation strength test. Also, they showed much higher ITS values than the others. This indicates that applying a higher PG to the base binder improves rutting resistance.

#### 3.2.3. TSR

**Figure 10:**
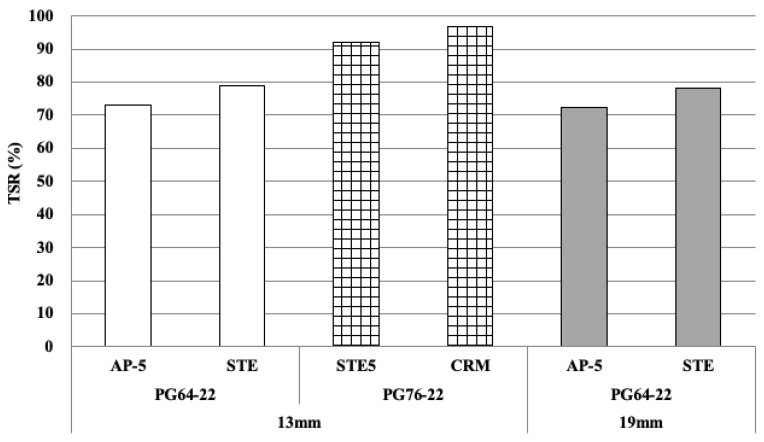
TSR results for 13 mm and 19 mm asphalt mixtures.

To measure the water sensitivity of the asphalt mixture, the tensile strength ratio (TSR) was measured, as shown in Figure 10. After the freeze–thaw test of general mixtures (PG64-22) without modifiers, the ratio was 73.0% and 72.2% for the maximum size of 13 and 19 mm aggregates, respectively. Accordingly, when modified with STE (PG64-22), the 13 mm and 19 mm asphalt mixtures exhibited TSR values of 78.9% and 78.0%, respectively. Notably, the PG76-22-modified binders (STE5 and CRM) achieved TSR values of 92.1% and 96.8% for the 13 mm aggregate size. Both mixtures exceeded 90%, indicating high resistance to moisture damage. When comparing PG64-22 for AP-5 and STE, the asphalt binder modified with STE showed a higher TSR compared to AP-5, indicating that STE modification effectively improves moisture resistance. In addition, even with the same PG grade, the moisture resistance of the asphalt mixture can be enhanced depending on the type of modifier. Also, using a stronger binder improves the mixture’s performance related to moisture.

#### 3.2.4. Wheel Tracking Test

The standard wheel tracking test for measuring the permanent deformation of asphalt mixtures involves assessing rut depth and dynamic stability using a reciprocating wheel motion for 60 min at a speed of 42 passes per minute at 60 °C. However, in this study, the test duration was extended to 180 min to better evaluate permanent deformation for railway applications. The asphalt mixture using AP-5 showed the largest rut depth at 12.2 mm, while the mixture of STE5 showed the smallest at 4.6 mm. When comparing the AP-5 and STE of PG64-22 with the CRM and STE of PG76-22 using 13 mm aggregate, a difference of about 7.05 mm was observed depending on the base binder’s performance grade. In addition, when comparing asphalt mixtures with the same performance grade, STE in the case of PG64-22 showed a smaller rut depth, with a difference of 1.3 mm compared to AP-5. In contrast, for PG76-22, the difference between the two binders was insignificant at 0.1 mm. This indicates that the effect of asphalt binder is significant on rutting resistance, as it compensates for the performance difference.

**Figure 11:**
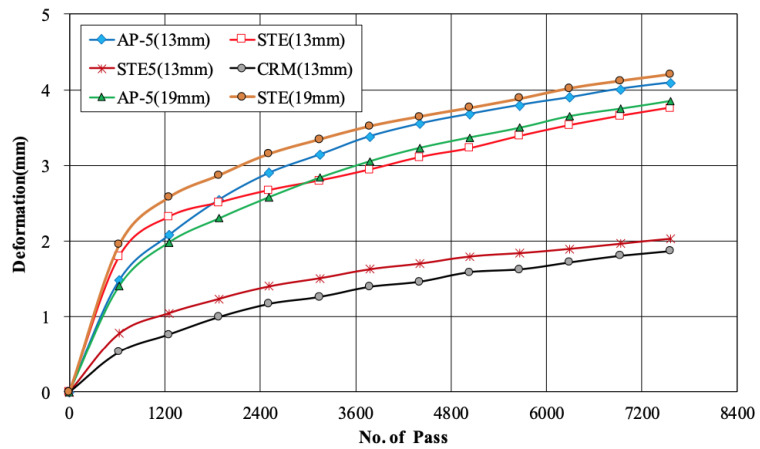
Graph of final deformation for each asphalt mixture.

**Figure 12:**
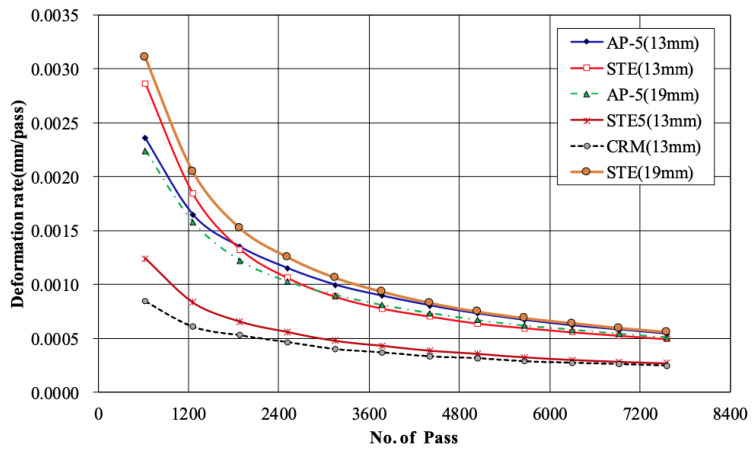
Rate of deformation by number of passes for each asphalt mixture.

In the 19 mm asphalt mixture, the STE was 1.3 mm smaller than that of AP-5. Comparing the difference in rut depth according to the aggregate size for these two binders that the rut depth for the 19 mm AP-5 was 1.8 mm higher than for the 13 mm asphalt mixture. Overall, it was confirmed that the type of binder had a more significant impact on wheel tracking rut depth than the aggregate size, as mixtures using the same binder exhibited similar results despite differences in their maximum aggregate size. The rut depth was graphed according to the pass for the six types of asphalt mixtures, as shown in Figure 11. A rut depth between 60 and 180 min was evaluated. There was almost a similar difference between asphalt mixtures and the same PG asphalt mixture, but a gap of about 3.1 mm was observed when the grades were different. Overall, the slope of the graph was steep between 630 passes and 1260 passes for all asphalt mixtures, showing that the deformation rate was similar to the deformation shown in Figure 12.

**Figure 13:**
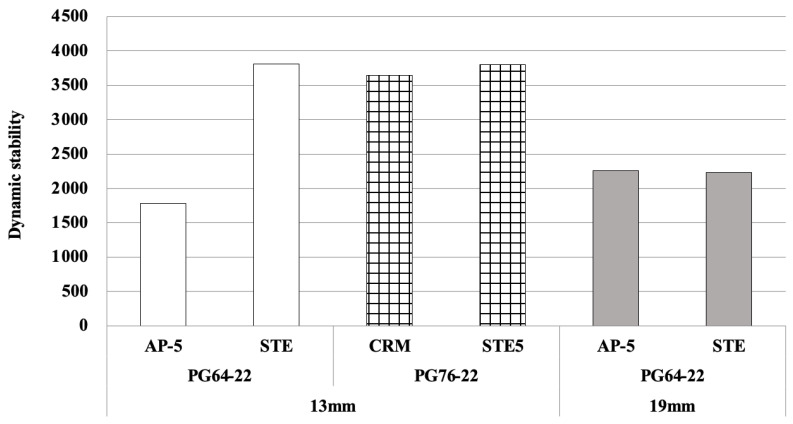
Results of the dynamic stability for each asphalt mixture.

The modified wheel tracking test is a method used to evaluate the resistance of mixtures to permanent deformation under dynamic loading. In this study, slab specimens for each mixture were made to calculate and assess the dynamic stability through final deformation after repeated running for 180 min (7560 times). The greater the dynamic stability used as an evaluation index in the repeated driving test, the smaller the rut depth and the better the permanent deformation resistance. As shown in Figure 13, the asphalt mixture using the PG76-22 binder (STE5 and CRM) showed the highest dynamic stability, whereas the asphalt mixture using the PG64-22 binder showed similar results, regardless of the mix design. From these results, it can be concluded that the grade of asphalt binder has a decisive influence on plastic deformation.

As a result, all mixtures produced with the four types of binders and two sizes of aggregate were tested for potential application in railroad infrastructure, as they met all the required performance criteria. This suggests that asphalt, commonly used as a pavement material, is also suitable for use in railroad systems.

## 4. Summary and Conclusions

This study evaluated the overall characteristics of asphalt binders and mixtures to improve the quality of asphalt concrete layers applied to railways. The properties of all four types of binders, including general asphalt, were analyzed. The test specimen for deformation strength, Marshall stability, and indirect tensile strength was prepared with the optimum asphalt content determined in the mix design. The deformation strength and Marshall stability at 60 °C and indirect tensile strength at 25 °C were evaluated. In addition, slab specimens were manufactured, and wheel tracking tests were performed at 60 °C. Based on these results, the following conclusions were drawn:

(1) As for rotational viscosity among the four asphalt binders (AP-5, STE, CRM, STE5), STE5 showed the highest viscosity of 1.8Pa·s, and CRM showed a value of 1.0Pa·s. AP-5 and STE showed a difference of 0.01P∙s in rotational viscosity, confirming no significant difference. The modified asphalt of CRM and STE5 had a lower penetration value and a higher softening point than AP-5 and STE. This is considered to be a phenomenon caused by the increased elasticity rather than viscosity of asphalt because CRM and STE5 have relatively high polymer content. Through this analysis, the Superpave binder tests were additionally performed and showed that each binder satisfied the standard for performance grade.
(2) Deformation strength was measured, showing an average of 4.44 MPa for the 13 mm mix design according to the maximum size of coarse aggregate in PG64-22 grade, whereas for the 19 mm mix design, the average result was 4.67 MPa. The analysis suggests that the optimum asphalt content is low, so the deformation is low due to the low flow of the asphalt binder, causing the deformation strength value to be slightly higher.
(3) Regarding ITS results, the PG76-22 asphalt mixture showed a higher value of about 0.33 MPa compared to the PG64-22 using the 13 mm mix design. The ITS values for both the 13 mm and 19 mm PG64-22 were similar, about 0.91 MPa on average. Also, the higher the PG grade, the higher the indirect tensile strength. Therefore, it is considered advantageous for the upper mixture, which is relatively vulnerable to the dynamic loads of the railway.
(4) After the freeze–thaw test of general mixtures (PG64-22) without modifiers, the TSR was 73.0% and 72.2% for the maximum size of 13 and 19 mm aggregates, respectively. Also, STE mixtures with 13 and 19 mm aggregates had TSR values of 78.9% and 78.0%. The modified asphalt (PG76-22), specifically the 13 mm asphalt mixture modified with STE5 and CRM, showed TSR values of 92.1% and 96.8%. This indicates that the STE and CRM modifiers are effective in improving resistance to moisture for railroad applications.
(5) Overall, in the wheel tracking test, the final rut depth of the 13 mm asphalt mixture was lower than that of the 19 mm mixture. In the case of the PG76-22 mixture, the final rut depth was more than twice as low as that of PG64-22. Based on these results, it is concluded that STE and CRM modifiers greatly affect plastic deformation resistance. It was also found that asphalt mixtures are suitable for use in railroad applications, as all measured rut depths remained below the maximum allowable limits.
(6) The asphalt mixtures prepared with four different binder types and two aggregate sizes successfully met all the required performance criteria, demonstrating their suitability for application in railroad infrastructure. These results highlight the potential of asphalt—traditionally employed in roadway pavement—as a durable and reliable material for railway systems, thereby expanding its utility beyond conventional road construction.
(7) This study evaluated the overall characteristics of asphalt mixtures based on road specification standards. Although all the results met or exceeded the standards, it is concluded that there is a need to conduct more in-depth research in the future, considering the various mechanical loads present in the railway.

## Figures and Tables

**Table 1:** Aggregate properties.

Classification		Apparent Specific Gravity (g/cm^3^)	Absorption (%)	Abrasion (%)
Specification		>2.5	<3.0	<40
Coarse agg.	13 mm	2.65	0.81	19.5
	19 mm	2.71	1.33	30.7
Fine agg.		2.67	1.01	-
Mineral filler		2.75	-	-

**Table 2:** Types of asphalt binders.

Base Binder	Binder	Description
PG64-22	AP-5	Straight asphalt
	STE	STE (1.5%)-modified asphalt mixture
PG76-22	STE5	STE (5%)-modified asphalt mixture
	CRM	CRM (8%)-modified asphalt mixture

**Table 3:** Description of the mixture.

Agg.	Max. Size	PG Grade	Mixture	Description
Granite	13 mm	PG64-22	AP	Granite 13 mm DGA grade, AP-5
			STE	Granite 13 mm DGA grade, STE 1.5%
		PG76-22	STE5	Granite 13 mm DGA grade, STE 5%
			CRM	Granite 13 mm DGA grade, CRM 8%
	19 mm	PG64-22	AP	Granite 19 mm DGA grade, AP-5
			STE	Granite 19 mm DGA grade, STE 1.5%

**Table 4:** Quality standard of asphalt mixtures for railway.

Material	Designation (Type, Grain Size, Layer Type)		Unit	ACT20MC	ACT13WC
Aggregate	Maximum aggregate size		mm	20	13
	Passing on sieve (%)	30 mm	%	–	–
		25 mm	%	100	–
		20 mm	%	95–100	100
		13 mm	%	80–92	95–100
		10 mm	%	70–85	83–92
		5 mm	%	48–65	55–70
		2.5 mm	%	30–50	35–50
		0.6 mm	%	15–30	18–30
		0.3 mm	%	11–23	13–22
		0.15 mm	%	7–15	8–15
		0.08 mm	%	4–10	4–10
Asphalt	Asphalt performance grade			PG64-22	PG76-22
	Number of gyratory compactions (Marshall compaction)		time	75 (50)	100 (75)
	Air void		%	2–4	1–3
	VFA		%	75–85	80–90
	VMA		%	≥12.0	≥12.0
	Minimum binder content		%	4.3	5.5
	Deformation strength (Marshall stability)		N (MPa)	≥3.2 (7000)	≥4.25 (8500)
	Flow value		1/100 cm	30–60	40–60
	TSR		%	≥0.7	≥0.8
	Dynamic stability		pass/mm	≥800	≥3000
	Permanent deformation		mm	≤8	≤3

**Table 5:** Test results of the physical properties of asphalt.

Property		PG64-22		PG76-22	
		AP-5	STE	CRM	STE5
Kinematic viscosity @135 °C (Pa∙s)		0.39	0.4	1.0	1.8
Softening point (°C)		46.4	47.5	63.0	68.5
Penetration (1/10 cm)		74	71	44	49
15 °C Ductility (cm)		>100	>100	65	59
G*/sin *δ*, kPa, (Temp.) @10 rad/s	Original	1.14 (64 °C)	1.31 (64 °C)	2.36 (76 °C)	1.81 (76 °C)
	RTFO	2.34 (64 °C)	2.82 (64 °C)	3.50 (76 °C)	3.10 (76 °C)
G*·sin *δ*, kPa, (Temp.) @ 10 rad/s	PAV	3250 (25 °C)	3540 (25 °C)	1820 (25 °C)	1680 (25 °C)
Creep stiffness (−12 °C)		155	160	170	155
*m*-value (−12 °C)		0.34	0.35	0.33	0.32

G*: The complex shear modulus of an asphalt binder.

**Table 6:** Properties of asphalt mixtures at OAC.

Agg. Size (mm)	PG	Binder	AP Cont. (%)	Density (g/cm^3^)	Air Void (%)	VMA (%)	VFA (%)
13	64-22	AP-5	5.2	2.423	2.47	14.80	83.31
		STE	5.1	2.422	2.62	14.71	82.18
	76-22	STE5	5.6	2.401	1.62	14.54	88.85
		CRM	5.6	2.375	2.61	15.62	83.31
19	64-22	AP-5	5.0	2.428	2.52	14.40	82.47
		STE	4.9	2.430	2.43	14.32	83.01

## Data Availability

The original contributions presented in this study are included in the article. Further inquiries can be directed to the corresponding authors.
